# Supplementary material for: Deficiency in endocannabinoid synthase DAGLB contributes to early onset Parkinsonism and murine nigral dopaminergic neuron dysfunction
Source: Nat Commun. 2022 Jun 17;13:3490. doi: 10.1038/s41467-022-31168-9 (PMC9205912; doi:10.1038/s41467-022-31168-9)
Supplement: Supplementary file 1 — Supplementary information [file 41467_2022_31168_MOESM1_ESM.pdf]

Deficiency in endocannabinoid synthase *DAGLB* contributes to early-onset Parkinsonism and murine nigral dopaminergic neuron dysfunction

Supplementary Materials:

1. **Supplementary Tables**
2. **Supplementary Figures**
3. **Additional AMP-PD and AMP-PD cohort acknowledgments**

Supplementary Table 1

Table S1. Homozygous segments larger than 2Mb identified in both affected sibling in Family 1 by homozygosity mapping through genome-wide SNP genotyping

No.	Chromosome	Start	End	Length (Kb)	SNP number	SNP density (SNP per kb)	Proportion of sites homozygous	Proportion of sites heterozygous
1	chr3	9244585	15423557	6179	2669	2.315	0.993	0
2	chr5	24837962	30738438	5900	1396	4.227	0.993	0
3	chr6	100815	2631241	2530	1151	2.198	0.995	0.003
4	chr7	1733102	8721760	6989	2674	2.614	0.999	0
5	chr17	30861865	34353274	3491	1419	2.46	0.994	0.001

Supplementary Table 2

Table S2. Whole-exome sequencing in three PD families

Family	Family 1		Family 2	Family 4
Family member	II-3	II-4	II-4	II-1
Total clean reads	87,161,148	82,704,614	80,568,752	63228822
Total clean data (Mb)	10849.79	10269.50	10021.30	9368.32
Mapped rate (%)	99.89	99.84	99.91	99.74
Mean depth	137.90	130.75	128.22	111.60
Coverage≥10X	99.0%	97.8%	99.1%	98.3%
Coverage≥20X	97.5%	95.1%	97.4%	99.5%
Coverage≥30X	94.1%	90.8%	93.9%	94.6%
Total variants	38837	37991	38454	39864
Nonsynonymous variants in exon or splicing (±2bp) region	11814	11357	11770	12273
MAF＜0.01 in East Asian population in GnomAD exome, GnomAD genome, and ExAC	547	557	577	597
Deleterious predicted by Reve	114	114	119	126
Homozygous variants in affected individuals	*DAGLB*		*DAGLB, OPLAH, HABP2, SSBP4*	*DAGLB*

Supplementary Table 3

Table S3. Identified mutations in *DAGLB* and predictions of their pathogenicity

Family	Family 1	Family 2	Family 4
Zygosity	homozygous	homozygous	homozygous
Chromosome Postion[a]	ch7: 6449668	ch7: 6464435	ch7: 6474600
cDNA alteration[b]	c.1821-2A>G	c.1088A>G	c.470dupC
Amino Acid Alteration	modify donor splice sites	p.D363G	p.L158Sfs*17
Exon function	Splicing	Nonsynonymous SNV	Frameshift insertion
MutationTaster[c]	Deleterious	Deleterious	Deleterious
CADD[c]	24.6	29.8	N/A
Reve[c]	Deleterious	Deleterious	Deleterious
gnomAD_exome_EAS[d]	absent	absent	absent
gnomAD_genome_EAS[d]	absent	absent	absent
ExAC_EAS[d]	absent	absent	absent
Chinese control cohort 1 (n=1,652, investigated by Whole-exome sequencing)[e]	absent	absent	absent
Chinese control cohort 2 (n=500, investigated by Sanger direct sequencing)[f]	absent	absent	absent

[a]Position on Genome Reference Consortium human genome build 37 (GRCh37)

[b]Accession number for *DAGLB* is NM_139179

[c]Mutation prediction by MutationTaseter, CADD and Reve

[d]Frequency of the mutation in East Asian population (from genomAD exome, genomeAD genome, and ExAC databases) was calculated by mutated allele number/total allele number in parentheses

[e]Allele frequencies of these pathogenic mutations in Han Chinese controls consisted of 1,652 healthy individuals detected by Whole-exome sequencing

[f]Allele frequencies of these pathogenic mutations in Han Chinese controls consisted of 500 healthy individuals detected by Sanger direct sequencing

Supplementary Table 4

Table S4. Clinical characteristics of patients with biallelic *DAGLB* mutations

	Family 1<br>II-3	Family 1<br>II-4	Family 2<br>II-4	Family 3<br>II-1	Family 3<br>II-2	Family 4<br>II-1
Mutation	c.1821-2A>G	c.1821-2A>G	c.1088A>G:<br>p.D363G	g.chr7:6,486,38<br>3-6,489,136 del	g.chr7:6,486,38<br>3-6,489,136 del	c.469dupC:<br>p.L158Sfs*17
Symptoms at onset	Resting tremor	Resting tremor	Bradykinesia	Resting tremor	Resting tremor	Bradykinesia
Asymmetry at onset	+	+	+	+	+	+
Hoehn-Yahr stage (Off/On)	IV/II	IV/II	IV/II	III/II	V/III	III/II
Motor symptom						
Bradykinesia	+	+	+	+	+	+
Resting tremor	+	+	+	+	+	+
Rigidity	+	+	+	+	+	+
Postural instability	+	+	+	+	+	+
UPDRS III (Off/On)	56/37	74/40	76/33	52/30	76/58	50/28
Nonmotor symptom						
Hypomimia	+	+	+	+	+	+
Depression	+	+	+	+	+	+
Urinary urgency	+	+	+	-	+	+
Constipation	-	+	+	-	+	+
Cognitive decline	-	-	-	+	-	-
Hallucination	+	-	-	-	-	-
Sleep disturbance	+	+	+	+	+	+
Freezing gait	+	+	+	+	+	+
RBD	-	+	+	+	-	-
Response to levodopa	+	+	+	+	+	+
Complications with						

treatment						
Wearing off	+	+	+	+	+	+
On-off phenomenon	+	+	+	+	+	+
Dyskinesia	+	+	+	-	-	+
Surgical therapies	NA	NA	+*	NA	+[#]	NA
Brain MRI	-	-	-	-	-	-
Brain ^{11}C-CFT PET	NA	NA	Abnormal[*]	NA	NA	NA

+ = Present; - = Absent; NA=Not performed

[*] Deep brain stimulation

[#] Posteroventral pallidotomy

[*]Severe striatal uptake deficit, particularly at putamen level, as seen in Parkinson's disease

MRI=magnetic resonance imaging; PET=positron emission tomography; CFT=C-2β-carbomethoxy-3β-(4-fluorophenyl) tropane

Supplementary Table 5

Table S5 List of primers used in this study

Splicing analysis

RT-PCR	Primer name	Sequence
c.1821-2A>G	RT-PCR Primer Forward	GATGTGATTCCCAGGCTCAG
	RT-PCR Primer Reverse	TCAGGCCACGTCCACACT

Breakpoint PCR

PCR	Primer name	Sequence
g.ch7:6,486,383-6,489,136del	Primer F1	CCAAAACAAGGCAAGGTTCACT
	Primer R1	CAAGTAGCCAGGACTACAGGTGC
	Primer F2	TCTCGGTTCAACACGCAAGCCCCT
	Primer R2	CTGCACCCTGCCTGGGACT
	Primer F3	CCAAAACAAGGCAAGGTTCACT
	Primer R3	GGGTCTCACTCTGCTACCCAGG

DAGLB direct sequencing

Primer name	Sequence
EXON1-F	GCAGACCTGCAATCGACTC
EXON1-R	CCACTTCTGTCACCGTCTCA

EXON2-F	GCCCTGTCCCCTTTTATTTC
EXON2-R	CGCCTGGTACAGGATTTCTT
EXON3-F	AAAGTCAGGAGGCGGGTG
EXON3-R	CGCAGAAACTAACTCCAATTTC
EXON4-F	CCTCGGTAACAGAACCCTCC
EXON4-R	CCACACACCCAAAGACACC
EXON5-F	CTGCCAGGAGCAGTCTTTTT
EXON5-R	GAGGGGAAAGGGGAATGA
EXON6-F	GAGAGTTCTATTCAACGAAGGAGC
EXON6-R	TACAGCGCATGTGACCAG
EXON7-F	AAAAGCCATTCCATGTCAGC
EXON7-R	TGGCCCCTCTGAAATAGTACAC
EXON8-F	CCCACTGTGTAGTGAGCGTG
EXON8-R	TCTGTCCTCACCATTTTCCC
EXON9-F	CGCTGGGTTTCCCTCTTTAG
EXON9-R	GCTCTGTGCAGTCCCTGAC
EXON10+11-F	GACGTGGCTGCTGATTCTG
EXON10+11-R	TACAGACACCCGCCAGCAT
EXON12+13-F	AACTGACGTTTCCCCTACCC
EXON12+13-R	TGATCAGATGGTGGAAGGAG
EXON14+15-F	GCATCTTTGCTGGAGTCTTCC
EXON14+15-R	GACCATGGAATTCTGTTCCC

Supplementary Figure 1

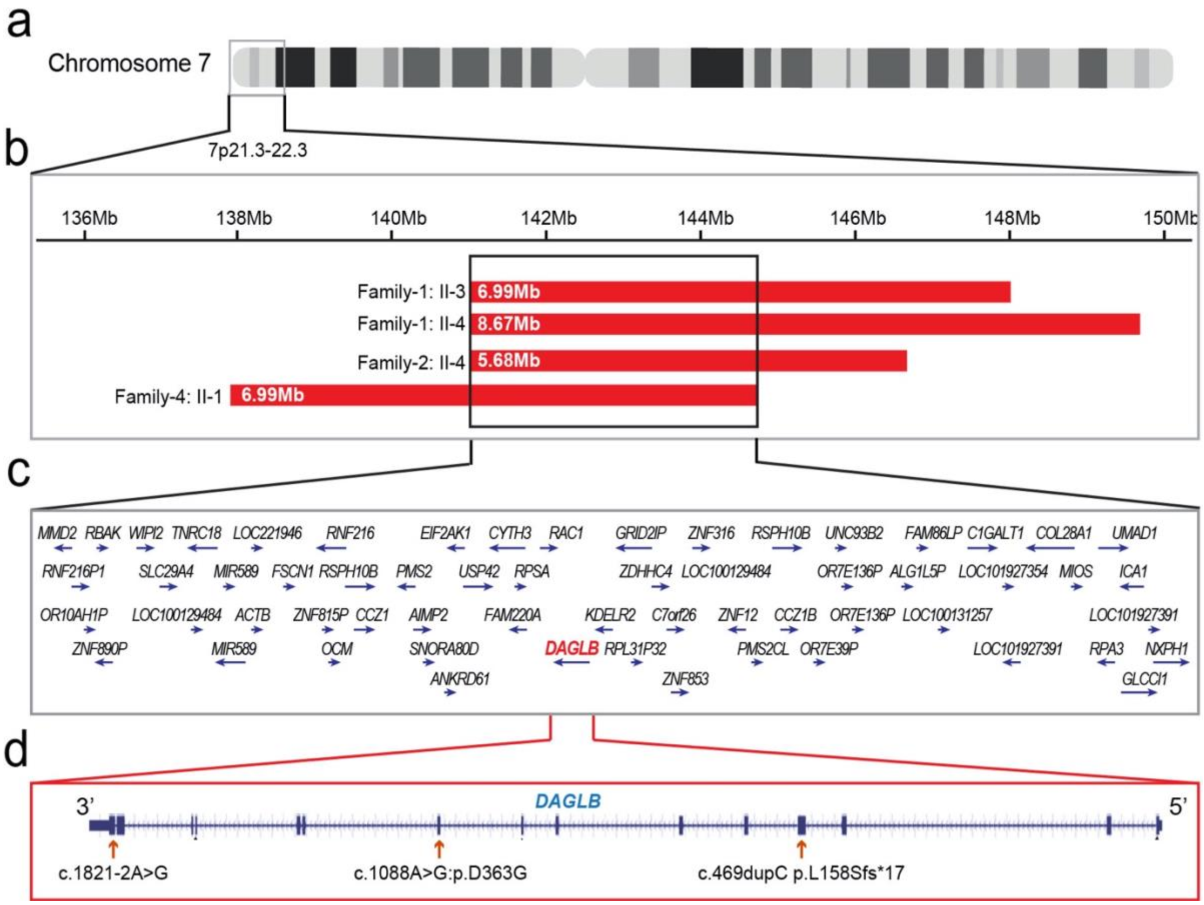

Supplementary Fig. 1 Molecular genetic findings in patients with *DAGLB* variants. (a) Schematic of chromosome 7. (b) Homozygosity mapping of affected families showing the overlapping regions of homozygosity on 7q21.3-7q22.3 (GRCh37/hg19). Run of homozygosity regions for each case are shown as red boxes. Black lines indicate the minimal overlap region. (c) Genes located in the overlap homozygosity region. (d) Schematic of the exon-intron structure of *DAGLB* indicating the positions of the frameshift insertion, missense mutation, and splice-site variant.

Supplementary Figure 2

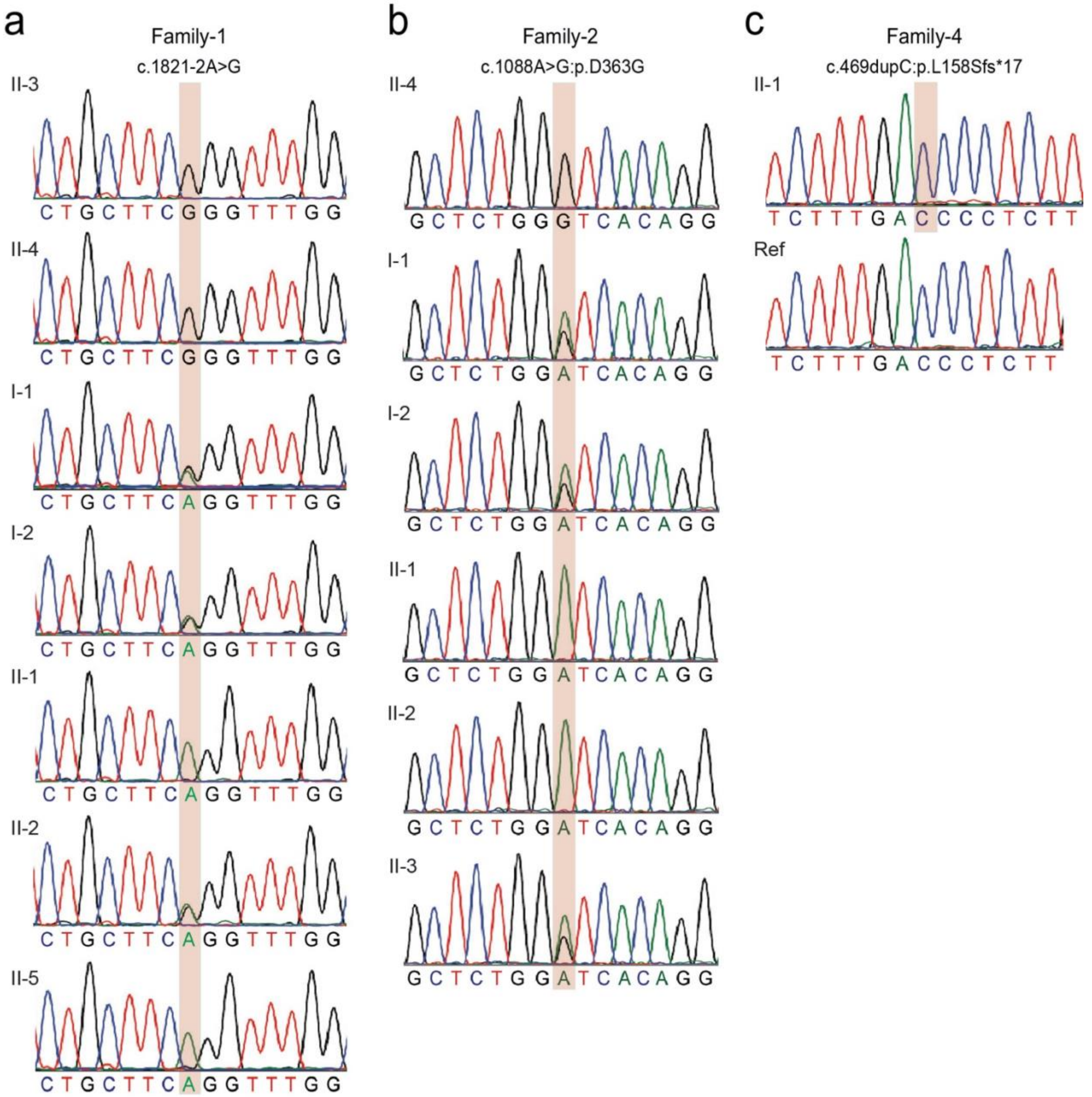

Supplementary Fig. 2 Sanger confirmation and segregation of the identified *DAGLB* variants. Sequence chromatograms of patients, their parents, and siblings from Family-1 **(**a), Family-2 (b), and Family-4 (c) (where available) are shown. Mutations were marked by pink box.

Supplementary Figure 3

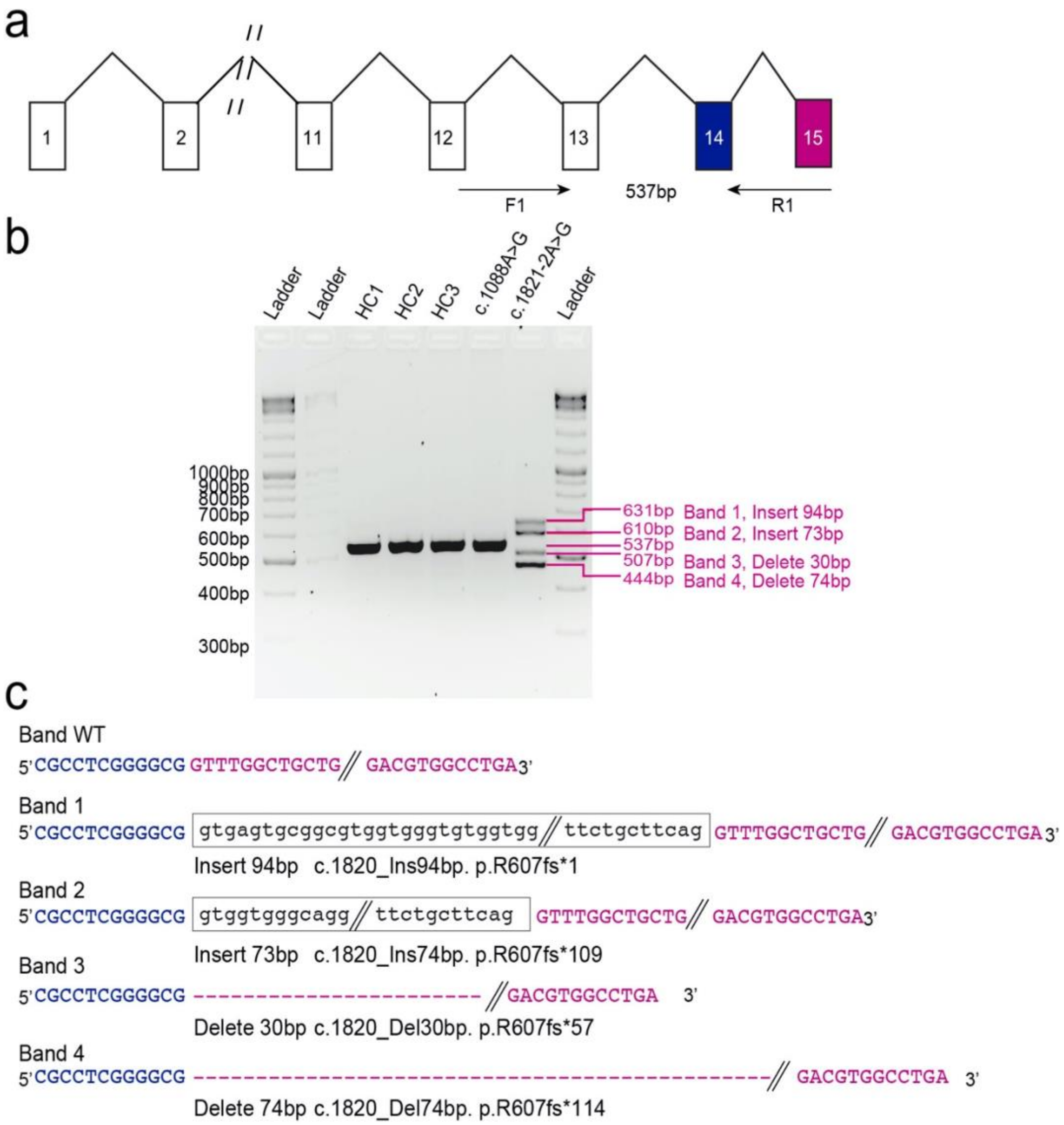

Supplementary Fig. 3 The c.1821-2A>G mutation resulted in aberrant splicing of *DAGLB*. (a) A partial gene structure for *DAGLB*. Exons are boxed. The primer pair (F1 and R1) used for amplification are shown as arrows below the exon. (b) Fibroblast RNA samples from the affected individuals (Family 1 II-3, Family 2 II-4) and three health control subjects demonstrated four bands (631bp, 610bp, 507bp and 444bp) for c.1821-2A>G variant (Family 1 II-3) but only one for other subjects. Three independent experiments were performed. (c) Gel purification, PCR and Sanger sequencing of the four bands from Family 1 II-3 demonstrate wild-type (WT) sequence and abnormal splicing sequence.

Supplementary Figure 4

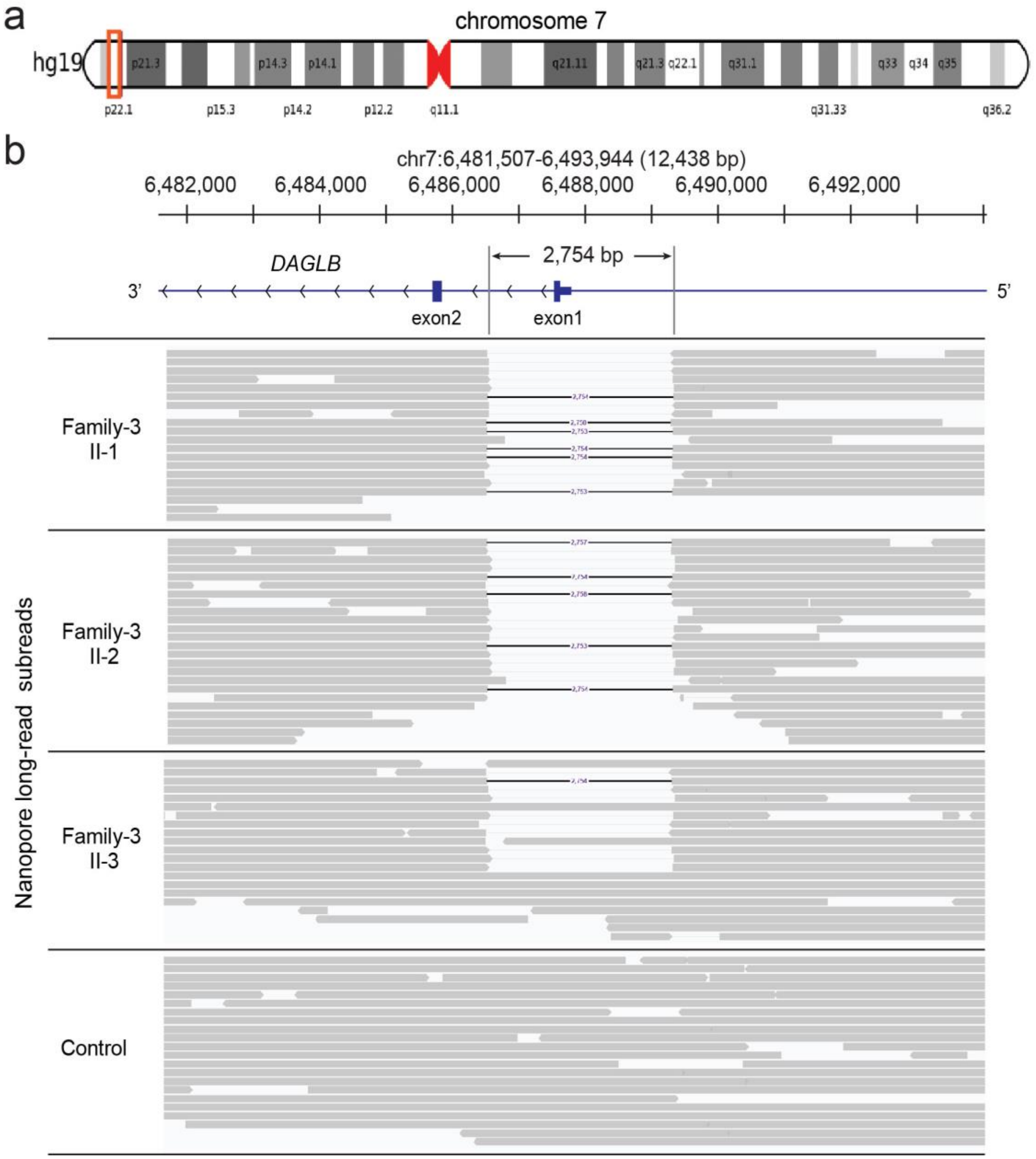

Supplementary Fig. 4 *DAGLB* deletion detected by Nanopore long-read sequencing. (a) Schematic of chromosome 7. (b) Nanopore long-read sequencing identifies a 2,754 bp deletion that includes the first coding exon of *DAGLB.* Nanopore long-read data were aligned to the human genome reference sequence (GRCh37/hg19). The site of deletion is shown by black connecting lines and subreads are shown by gray boxes. Nanopore long read subreads are shown for control, health sibling and the affected sisters from the Family 3. A homozygous 2,754 bp deletion involving *DAGLB* was supported by 20 or 24 Nanopore long reads without any read supporting the reference allele in the affected individuals (Family 3 II-1 and II-2) respectively, and 11 of 21 reads at the locus support the heterozygous deletion in the carrier sibling (Family 3 II-3).

Supplementary Figure 5

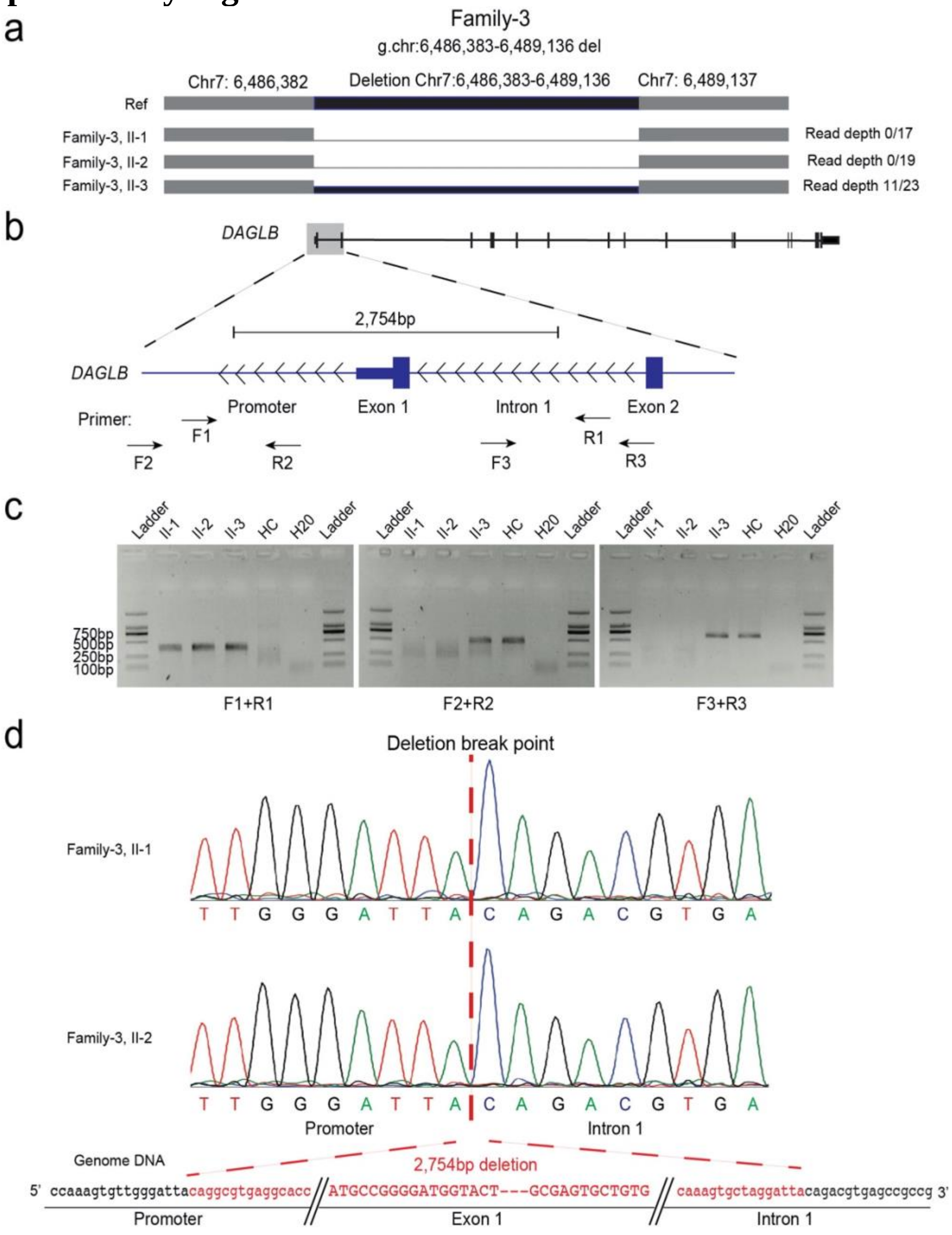

Supplementary Fig. 5 Characterization of a 2.7-kb deletion at the *DAGLB* locus. (a) The high-quality Nanopore long-reads identify a 2,754 bp deletion (chr7:6,486,383-6,489,136) involving *DAGLB*. (b) Using positional information from Nanopore calls, PCR primers were designed to amplify the breakpoint junction. (c) The junction fragment was amplified when using DNA from the affected individuals (homozygous deletion) and the carrier sibling (heterozygous deletion), but not from control DNA. Three independent experiments were performed. (d) This deletion variant was validated by Sanger sequencing, confirming the precise breakpoints identified by long reads sequencing.

Supplementary Figure 6

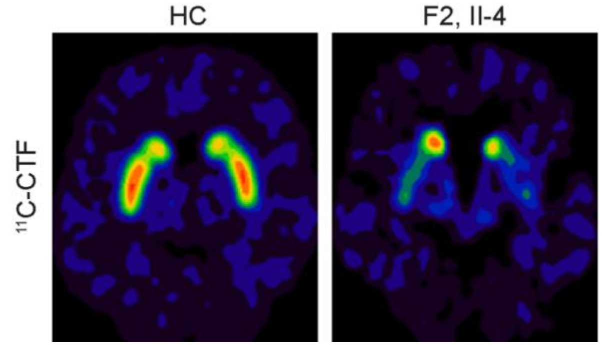

Supplementary Fig. 6 PET imaging of PD patient with *DAGLB* mutation. Representative axial PET images of ^{11}C-2β-carbomethoxy-3β-(4-fluorophenyl) tropane (^{11}C-CFT) uptake in an affected member (Family 2) and healthy control (HC), showing a graded and asymmetrical reduction in dopamine transporter binding (^{11}C-CFT) in the putamen.

Supplementary Figure 7

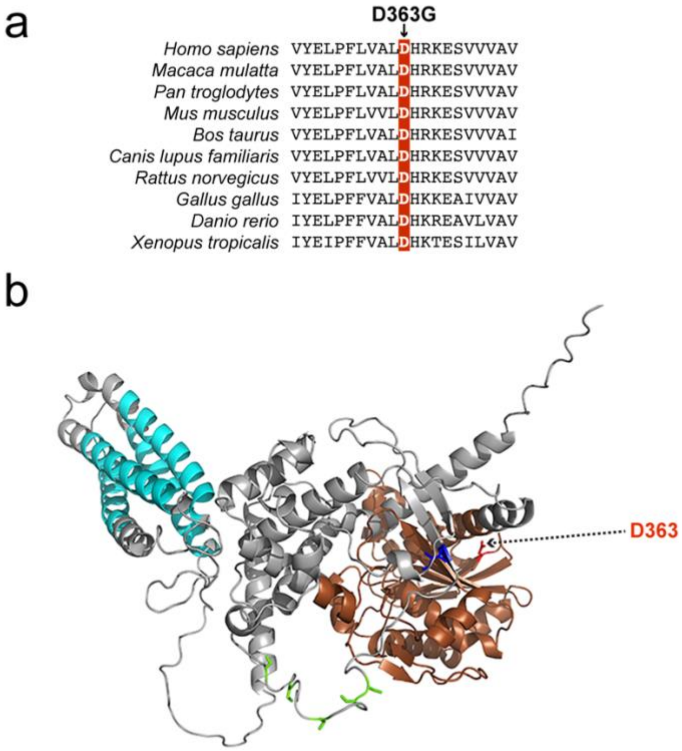

Supplementary Fig. 7 The conserved D363 residue in the catalytic domain of DAGLB across different species. (a) Sequence alignment. (b) 3D model of human DGLB protein constructed using molecular coordinates from computational predictions by AlphaFold. The N-terminal four transmembrane region regions were marked in cyan, and the conserved lipase domain was marked in brown. D363 was marked in red.

Supplementary Figure 8

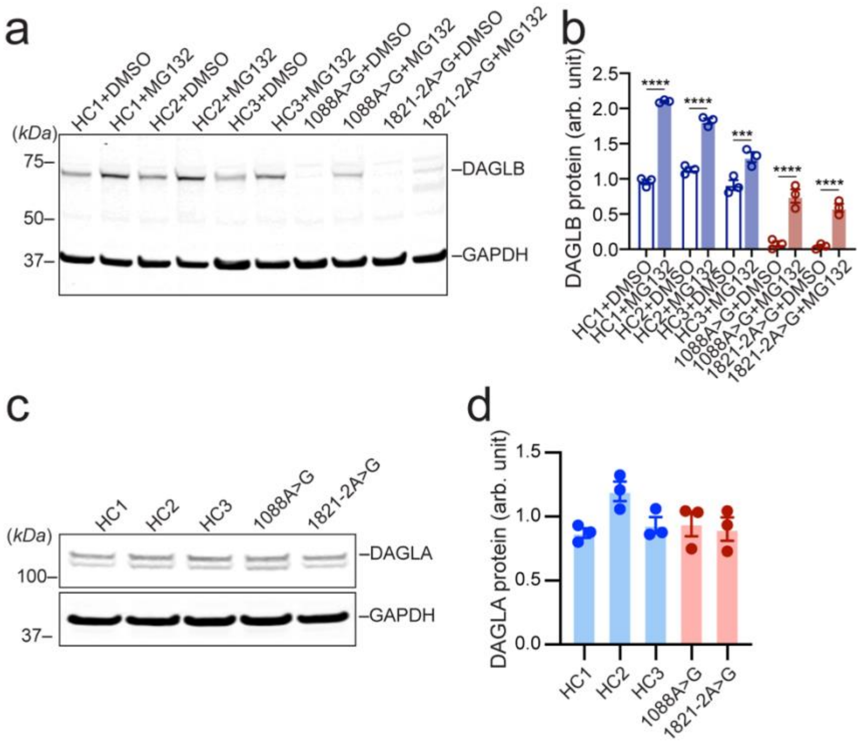

Supplementary Fig. 8 Proteasome inhibitor MG132 increased the levels of DAGLB protein in patient-derived fibroblasts. (a) Representative western blot and (b) quantification of protein levels of DAGLB in patient-derived fibroblast cells treated with vehicle or proteasome inhibitor MG132. Data were normalized to glyceraldehyde 3-phosphate dehydrogenase (GAPDH) protein. All experiments were performed three times, blinded to genotype (data represent mean ± SEM) 1-way ANOVA with Sidak's multiple comparison test, adjusted $^{***}p=0.0002$, $^{****}p<0.0001$. (c) Representative western blot and quantification (d) of DAGLA protein levels in patient-derived fibroblast cells. All experiments were performed three times, blinded to genotype (data represent mean ± SEM). Data were normalized to GAPDH protein.

Supplementary Figure 9

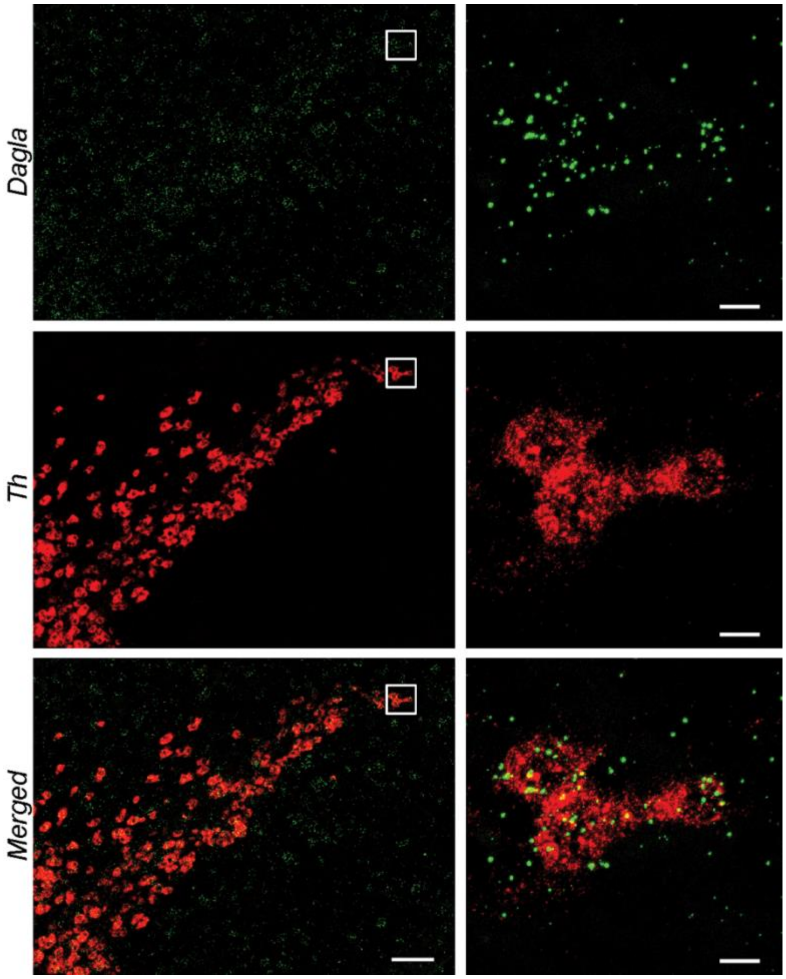

Supplementary Fig. 9 RNAscope *in situ* hybridization of *Dagla* and *Th* in mouse midbrain sections. Right panels highlight the boxed areas in the left panels. Scale bars: 100 μm (left) and 20 μm (right). More than five independent experiments were performed.

Supplementary Figure 10

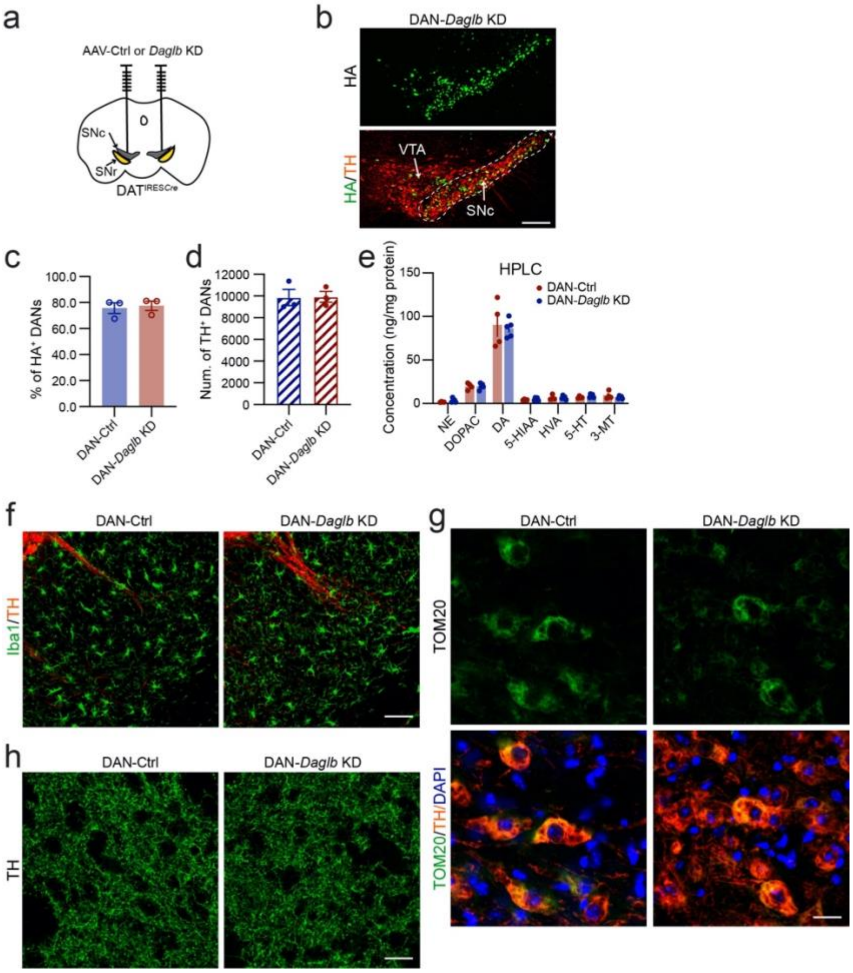

Supplementary Fig. 10 Pathological characterization of DAN-*Daglb* KD mice. (a) Bilateral stereotactic injection of AAV-control and AAV-*Daglb* KD vectors in the SNc of *Dat*$^{IRES cre}$ mice. (b) Representative images of HA-SaCas9 (green) and TH (red) staining. Scale bar: 200μm. More than 10 independent experiments were performed. (c) Percentages of HA-SaCas9-postive nigral DANs in the control and KD mice 4-5 months after AAV injection. N=3 per genotype, unpaired t test, two-tailed, p=0.75. (d) Numbers of TH-positive nigral DANs in the control and KD mice 12 months after AAV injection. N=3 per genotype, unpaired t test, two-tailed, p=0.94. (e) HPLC quantification of norepinephrine (NE), 3,4-Dihydroxyphenylacetic acid (DOPAC), dopamine (DA), 5-Hydroxyindoleacetic acid (5-HIAA), Homovanillic acid (HVA), 5-hydroxytryptamine (5-HT), and 3-Methoxytyramine (3-MT) in the dorsal striatum of DAN-Daglb KD (n=5) and control mice (n=4) 4-6 months after AAV injection. Multiple t-test, no significant group difference. All experiments were performed blinded to genotype. Data represents mean ± SEM. (f) Sample images of Iba1 (green) and TH (red) staining in the SNr of DAN-Ctrl and DAN-Daglb KD mice 4-6 months after stereotaxic surgery. Scale bar: 50μm. (g) Sample images of mitochondrial maker TOM20 (green) and TH (red) staining in the SNc of DAN-Ctrl and DAN-Daglb KD mice at 12 months of age. Scale bar: 20μm. (h) Sample images of TH staining in the dorsal striatum of DAN-Ctrl and DAN-Daglb KD mice at 4-6 months 4-6 months after stereotaxic surgery. Scale bar: 20μm.

Supplementary Figure 11

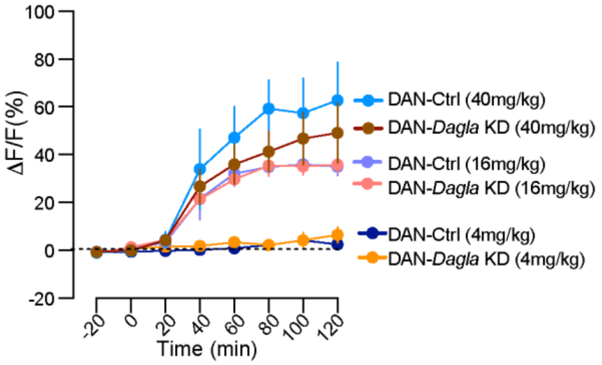

Supplementary Fig. 11 DAGLA does not contribute to the major 2-AG synthesis in nigral DANs. Time course of eCB2.0 signals in the SN of DAN-control and DAN-*Dagla* KD mice before and after treated with JZL184 at 4, 16, and 40 mg/kg. N=4 mice per genotype. Data were presented as mean ± SEM.

Supplementary Figure 12

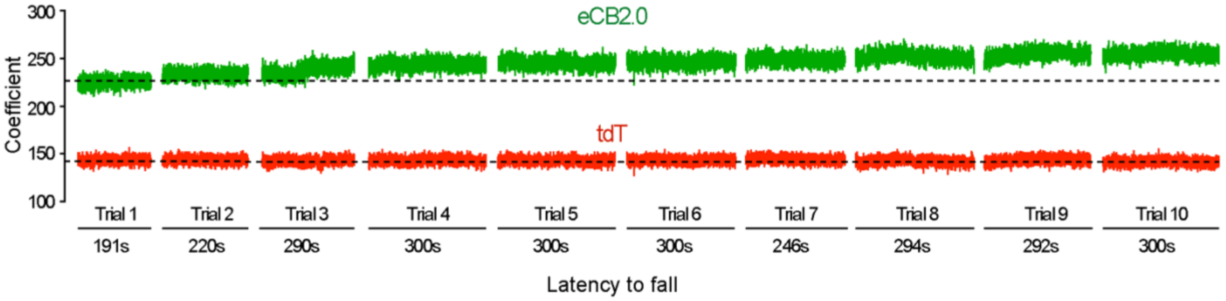

Supplementary Fig. 12 Progressive increase of 2-AG signals during rotarod motor skill learning. Representative raw data of eCB2.0 (green) and tdT (red) signals in the SNr of a mouse performing the 10-trial rotarod motor learning test on day 2 of the 6-day training paradigm.

Supplementary Figure 13

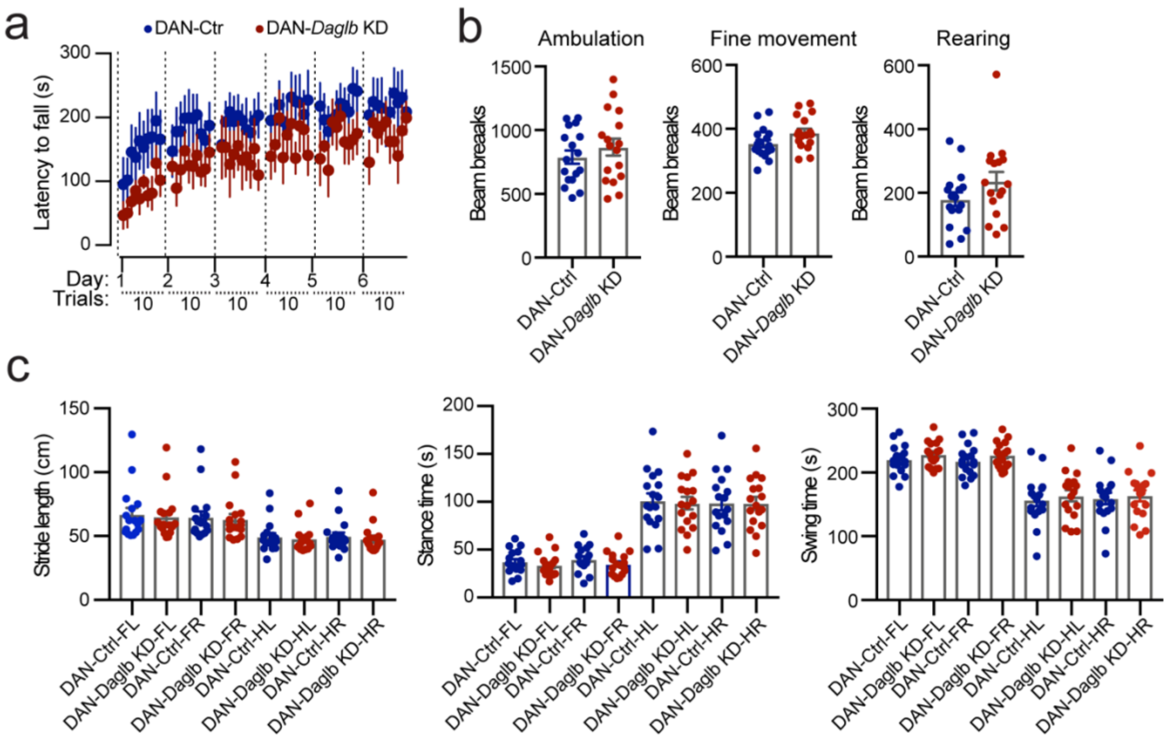

Supplementary Fig. 13 Normal locomotion and gait properties of DAN-*Daglb* KD mice. (a) Rotarod motor skill learning tests of DAN-Ctrl and DAN-*Daglb* KD mice (n= 5M per genotype) in main Fig. 5A. Data represent mean ± SEM. (b) Open-field tests show comparable travel distance (p=0.23), ambulatory (p=0.30), fine movement (p=0.06), and rearing (p=0.13) between 4-5-month-old *Daglb* control (n=17) and KD (n=17) mice. Data represent mean ± SEM, unpaired t test, two-tailed. (c) Gait analyses depict comparable stride length [front left limb (FL): p=0.76, front right limb (FR): p=0.77, hind left limb (HL): p=0.68, hind right limb (HR): p=0.58], stance time (FL: p=0.38, FR: p=0.26, HL: p=0.79, HR: p=0.98) and swing time (FL: p=0.29, FR: p=0.24, HL: p=0.60, HR: p=0.73) of 5-month-old *Daglb* control (n=17) and KD (n=17) mice. Data represent mean ± SEM, unpaired *t* test, two-tailed.

Supplementary Figure 14

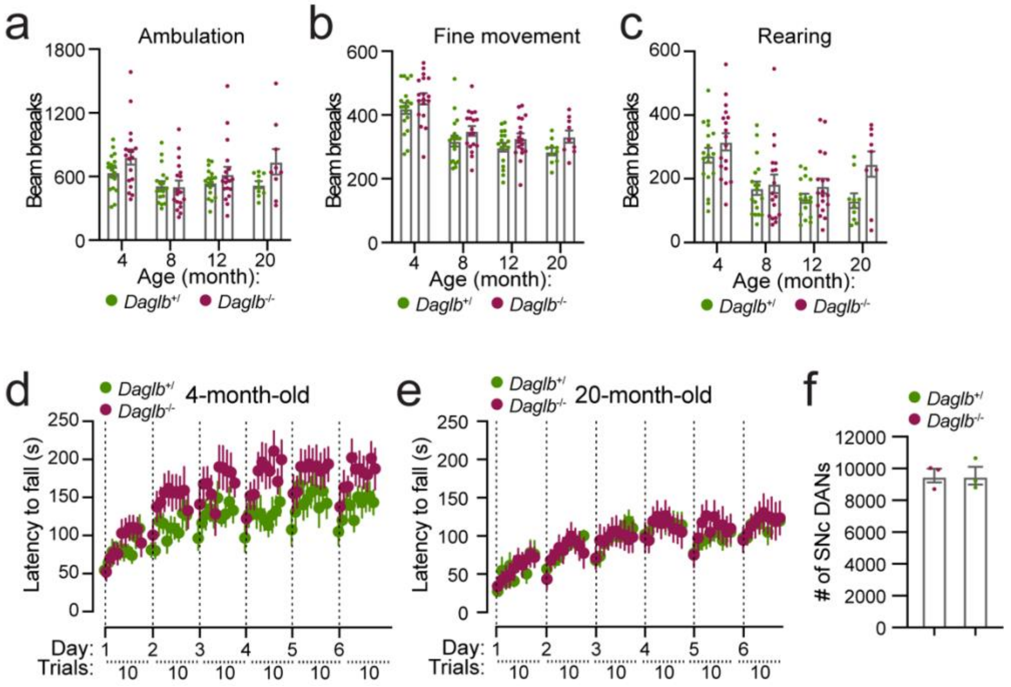

Supplementary Fig. 14 Behavioral and neuropathological characterization of *Daglb* germline KO mice. (a-c) Open-field tests show comparable ambulatory (a), fine movement (b), and rearing (c) between the control wild-type and heterozygous *Daglb* KO mice (*Daglb*$^{+/}$) at 4 (n=18), 8 (n=18), 12 (n=17), and 20 (n=10) months of age and age-matched homozygous *Daglb* KO (*Daglb*$^{-/-}$, n=18, 18, 18, 9) mice. Data represent mean ± SEM, unpaired t test. (d-e) Rotarod motor learning tests of *Daglb*$^{+/}$ (n=11, 6M5F) and *Daglb*$^{-/-}$ (n=10, 5M5F) mice at 4 (d) and 20 (e) months of age. Data represent mean ± SEM. (f) Numbers of TH-positive nigral DANs in 20-month-old *Daglb*$^{+/+}$ and *Daglb*$^{-/-}$ mice. N=3 per genotype. Data represent mean ± SEM. Unpaired *t* test, p=0.9989.

AMP PD Acknowledgement

The AMP® PD program is a public-private partnership managed by the Foundation for the National Institutes of Health and funded by the National Institute of Neurological Disorders and Stroke (NINDS) in partnership with the Aligning Science Across Parkinson's (ASAP) initiative; Celgene Corporation, a subsidiary of Bristol-Myers Squibb Company; GlaxoSmithKline plc (GSK); The Michael J. Fox Foundation for Parkinson's Research; Pfizer Inc.; Sanofi US Services Inc.; and Verily Life Sciences. “ACCELERATING MEDICINES PARTNERSHIP and AMP are registered service marks of the U.S. Department of Health and Human Services.

AMP PD Cohort Acknowledgements

The Clinical data and biosamples used in preparation of this article were obtained from the (i) Michael J. Fox Foundation for Parkinson’s Research (MJFF) and National Institutes of Neurological Disorders and Stroke (NINDS) BioFIND study, (ii) Harvard Biomarkers Study (HBS), (iii) National Institute on Aging (NIA) International Lewy Body Dementia Genetics Consortium Genome Sequencing in Lewy Body Dementia Case-control Cohort (LBD), (iv) MJFF LRRK2 Cohort Consortium (LCC), (v) NINDS Parkinson's Disease Biomarkers Program (PDBP), (vi) MJFF Parkinson’s Progression Markers Initiative (PPMI), and (vii) NINDS Study of Isradipine as a Disease-modifying Agent in Subjects With Early Parkinson Disease, Phase 3 (STEADY-PD3) and (viii) the NINDS Study of Urate Elevation in Parkinson’s Disease, Phase 3 (SURE-PD3).

i. BioFIND is sponsored by The Michael J. Fox Foundation for Parkinson’s Research (MJFF) with support from the National Institute for Neurological Disorders and Stroke (NINDS). The BioFIND Investigators have not participated in reviewing the data analysis or

content of the manuscript. For up-to-date information on the study, visit michaeljfox.org/news/biofind.

ii. Genome sequence data for the Lewy body dementia case-control cohort were generated at the Intramural Research Program of the U.S. National Institutes of Health. The study was supported in part by the National Institute on Aging (program #: 1ZIAAG000935) and the National Institute of Neurological Disorders and Stroke (program #: 1ZIANS003154).

iii. The Harvard NeuroDiscovery Biomarker Study (HBS) is a collaboration of HBS investigators [full list of HBS investigator found at https://www.bwhparkinsoncenter.org/biobank/ and funded through philanthropy and NIH and Non-NIH funding sources. The HBS Investigators have not participated in reviewing the data analysis or content of the manuscript.

iv. Data used in preparation of this article were obtained from The Michael J. Fox Foundation sponsored LRRK2 Cohort Consortium (LCC). The LCC Investigators have not participated in reviewing the data analysis or content of the manuscript. For up-to-date information on the study, visit https://www.michaeljfox.org/biospecimens).

v. PPMI is sponsored by The Michael J. Fox Foundation for Parkinson's Research and supported by a consortium of scientific partners: [list the full names of all of the PPMI funding partners found at https://www.ppmi-info.org/about-ppmi/who-we-are/study-sponsors]. The PPMI investigators have not participated in reviewing the data analysis or content of the manuscript. For up-to-date information on the study, visit www.ppmi-info.org."

vi. The Parkinson's Disease Biomarker Program (PDBP) consortium is supported by the National Institute of Neurological Disorders and Stroke (NINDS) at the National Institutes of

Health. A full list of PDBP investigators can be found at https://pdbp.ninds.nih.gov/policy. The PDBP investigators have not participated in reviewing the data analysis or content of the manuscript.

vii. The Study of Isradipine as a Disease-modifying Agent in Subjects With Early Parkinson Disease, Phase 3 (STEADY-PD3) is funded by the National Institute of Neurological Disorders and Stroke (NINDS) at the National Institutes of Health with support from The Michael J. Fox Foundation and the Parkinson Study Group. For additional study information, visit https://clinicaltrials.gov/ct2/show/study/NCT02168842. The STEADY-PD3 investigators have not participated in reviewing the data analysis or content of the manuscript.

viii. The Study of Urate Elevation in Parkinson's Disease, Phase 3 (SURE-PD3) is funded by the National Institute of Neurological Disorders and Stroke (NINDS) at the National Institutes of Health with support from The Michael J. Fox Foundation and the Parkinson Study Group. For additional study information, visit https://clinicaltrials.gov/ct2/show/NCT02642393. The SURE-PD3 investigators have not participated in reviewing the data analysis or content of the manuscript.
