# Supplementary material for: Deficiency in endocannabinoid synthase DAGLB contributes to early onset Parkinsonism and murine nigral dopaminergic neuron dysfunction
Source: Nat Commun. 2022 Jun 17;13:3490. doi: 10.1038/s41467-022-31168-9 (PMC9205912; doi:10.1038/s41467-022-31168-9)
Supplement: Supplementary file 2 — Reporting Summary [file 41467_2022_31168_MOESM2_ESM.pdf]

nature portfolio

Corresponding author(s): Drs. Huaibin Cai and Beisha Tang

Last updated by author(s): Apr 16, 2022

Reporting Summary

Nature Portfolio wishes to improve the reproducibility of the work that we publish. This form provides structure for consistency and transparency in reporting. For further information on Nature Portfolio policies, see our Editorial Policies and the Editorial Policy Checklist.

Statistics

For all statistical analyses, confirm that the following items are present in the figure legend, table legend, main text, or Methods section.

n/a	Confirmed	
☐	☒	The exact sample size (n) for each experimental group/condition, given as a discrete number and unit of measurement
☐	☒	A statement on whether measurements were taken from distinct samples or whether the same sample was measured repeatedly
☐	☒	The statistical test(s) used AND whether they are one- or two-sided<br>*Only common tests should be described solely by name; describe more complex techniques in the Methods section.*
☒	☐	A description of all covariates tested
☒	☐	A description of any assumptions or corrections, such as tests of normality and adjustment for multiple comparisons
☐	☒	A full description of the statistical parameters including central tendency (e.g. means) or other basic estimates (e.g. regression coefficient) AND variation (e.g. standard deviation) or associated estimates of uncertainty (e.g. confidence intervals)
☐	☒	For null hypothesis testing, the test statistic (e.g. F, t, r) with confidence intervals, effect sizes, degrees of freedom and P value noted<br>*Give P values as exact values whenever suitable.*
☒	☐	For Bayesian analysis, information on the choice of priors and Markov chain Monte Carlo settings
☒	☐	For hierarchical and complex designs, identification of the appropriate level for tests and full reporting of outcomes
☒	☐	Estimates of effect sizes (e.g. Cohen's d, Pearson's r), indicating how they were calculated

Our web collection on statistics for biologists contains articles on many of the points above.

Software and code

Policy information about availability of computer code

Data collection	1. PAS software (San Diego Instruments) was used to trace and quantify mouse movement in the unit as the number of beam breaks per 30 min.<br>2. Endocannabinoids were quantified by using Mass-Hunter Workstation LC/QQQ Acquisition and MassHunter Workstation Quantitative Analysis software (Agilent Technologies).<br>3. For fiber photometry recording, the measured emission spectra of eCB2.0, DA2m, GCaMP6f and tdTomato signals were fitted using a linear unmixing algorithm (https://www.niehs.nih.gov/research/atniehs/labs/ln/pi/iv/tools/index.cfm).The coefficients of eCB2.0, DA2m, GCaMP6f and tdTomato signals generated by the unmixing algorithm were used to represent the fluorescence intensities of eCB2.0, DA2m, GCaMP6f and tdTomato, respectively. To correct for movement-induced artifacts, the ratios of eCB2.0, DA2m or GCaMP6f signal intensities against the corresponding tdTomato signal intensities were used to represent the final normalized signal intensities.<br>4. Real-time Taqman PCR was performed on an ABI 7900HT with TaqMan Gene Expression Assays (Applied Biosystems, Life Technologies, Carlsbad, CA) for human DAGLB exon 9-10 (Hs00373700_m1).<br>5. For the western blot, the protein bands of interest were visualized with Odyssey CLx Infrared Imaging Studio. The band intensity was quantified using ImageJ.<br>6. For tissue staining, the fluorescent images were acquired using a laser scanning confocal microscope LSM 780 (Zeiss) with Zen software.<br>7. For RNA-sequencing, the libraries were then qualified using the Bioanalyzer DNA 1000 assay (Agilent) and sequenced with Illumina HiSeq 2000. The standard Illumina pipeline was used to generate Fastq files.
Data analysis	1. Homozygosity mapping was performed with PLINK (http://pngu.mgh.harvard.edu/purcell/plink/) for the identification of regions of homozygosity in affected individuals, and the minimum length for homozygous runs was set to 2 Mb.<br>2. The Picard tool (http://broadinsti tute.github.io/picard/) was used to remove duplicate reads, generate the converse format, and index the sequencing data.<br>3.Base quality-score recalibration, local realignments around possible insertions/deletions (indels), variant calling, and filtering were performed with the Genome Analysis Toolkit (GATK).

4. ANNOVAR was used to annotate single nucleotide variants and insertions/deletions with RefSeq (UCSC hg19).
5. The detection of copy-number variant (CNV) in DAGLB from WES data in our ARPD and sporadic EOPD cohorts was performed with the eXome-Hidden Markov Model (XHMM) software.The average baseline signals were calculated as FB. The instant signals at different time point after drug treatment were calculated as FI. The alterations of signal intensities at different time points were calculated as ΔF/F=(FI-FB)/FB.
6. Long-read nanopore sequencing reads were aligned to the human reference genome (hg19) using NGMLR (ngmlr 0.2.7), and structural variation calls were detected by Sniffles.
7.The Ensembl annotated transcript abundance was quantified using Salmon in a non-alignment-based mode, and gene level counts were estimated using Tximport package (Bioconductor). The counts for the resulting genes were then normalized using a variance-stabilizing transformation.
8. PD variants in AMP-PD dataset were classified into 2 classes (missense and LOF) as annotated using ANNOVAR (2020-06-08). PLINK was used to extract missense and LOF (splicing, stop, frameshift) variants at a MAF of 0.05, and to run genotypic disease-variant association testing, using the --model flag. After recoding the data into an additive model, summary statistics were extracted using R (version 4.1.0). Finally, RVtests 69 (v2.1) was used to run the CMC, CMC Wald, and SKAT-O burden tests for each of the defined variant classes at MAF of 0.05.
9. FreewalkScanTM2.0 software (CleverSys Inc) for various characteristic parameters of gait including stride length and stance/swing time of each paw.
10. TH-positive neurons were assessed using the fractionator function of Stereo Investigator 10 (MBF Bioscience).
11. In fiber photometry studies, the average baseline signals were calculated as FB. The instant signals at different time point after drug treatment were calculated as FI. The alterations of signal intensities at different time points were calculated as ΔF/F=(FI-FB)/FB.
12. All the data were analyzed by Prism 8 software (Graphpad).

For manuscripts utilizing custom algorithms or software that are central to the research but not yet described in published literature, software must be made available to editors and reviewers. We strongly encourage code deposition in a community repository (e.g. GitHub). See the Nature Portfolio guidelines for submitting code & software for further information.

Data

Policy information about availability of data

All manuscripts must include a data availability statement. This statement should provide the following information, where applicable:

- Accession codes, unique identifiers, or web links for publicly available datasets
- A description of any restrictions on data availability
- For clinical datasets or third party data, please ensure that the statement adheres to our policy

1. Study participants were recruited at Xiangya Hospital, Central South University between October 2006 and January 2019 and other hospitals of Parkinson's Disease and Movement Disorders Multicenter Database and Collaborative Network in China (PD-MDCNC, http://pd-mdcnc.com:3111/).
2. Mutation analysis of DAGLB in another 500 matched control cohort was done by direct sequencing (GenBank, NM_139179.4 and NP_631918.3).
3. The human DAGLA, DAGLA, and TH RNA sequencing dataset was obtained from GSE76514.
4. The accession number of the striatal tissue RNA-seq is PRJNA612478 (https://www.ncbi.nlm.nih.gov/bioproject/?term=prjna612478).
5. The accession number of SNc RNA-seq data is PRJNA775656 (https://www.ncbi.nlm.nih.gov/bioproject/775656).
6. The whole genome sequencing (WGS) data from the Accelerating Medicines Partnership - Parkinson's disease initiative (AMP-PD, https://amp-pd.org/) which includes data from multiple cohorts of unrelated European descent.

Field-specific reporting

Please select the one below that is the best fit for your research. If you are not sure, read the appropriate sections before making your selection.

☒ Life sciences ☐ Behavioural & social sciences ☐ Ecological, evolutionary & environmental sciences

For a reference copy of the document with all sections, see nature.com/documents/nr-reporting-summary-flat.pdf

Life sciences study design

All studies must disclose on these points even when the disclosure is negative.

Sample size	The sample size was estimated based on previous experiences. In general, three or more independent samples per group were used for western blot, qRT-PCR, histology, and fiber photometry recording. 10 or more mice with mixed genders were used for behavioral studies.
Data exclusions	No data were excluded.
Replication	The western blot, RT-PCR, cell counting, and fiber photometry recoding experiments were performed independently by two or more investigators. The results were consistent between the different investigators. The behavioral tests with a large cohort of mice and LCM-RNA sequencing were not replicated due to cost and time restrictions.
Randomization	The samples for western blot and qRT-PCR experiments were fully randomized. The samples for cell counting, fiber photometry recording and behavioral experiments were grouped based on age and gender balance. Within each group, the selection of individual animal was totally randomized.
Blinding	The investigators were blinded to genotypes when carrying out any experiments.

Reporting for specific materials, systems and methods

We require information from authors about some types of materials, experimental systems and methods used in many studies. Here, indicate whether each material, system or method listed is relevant to your study. If you are not sure if a list item applies to your research, read the appropriate section before selecting a response.

Materials & experimental systems

n/a	Involved in the study
☐	☒ Antibodies
☐	☒ Eukaryotic cell lines
☒	☐ Palaeontology and archaeology
☐	☒ Animals and other organisms
☐	☒ Human research participants
☒	☐ Clinical data
☒	☐ Dual use research of concern

Methods

n/a	Involved in the study
☒	☐ ChIP-seq
☒	☐ Flow cytometry
☒	☐ MRI-based neuroimaging

Antibodies

Antibodies used	Rabbit monoclonal anti-DAGLB (Cell Signaling, 12574S, clone D4P7C, 1:500), Rabbit polyclonal anti-DAGLA (Frontier Institute co. ltd, DGLa-Rb-Af380, 1:250), Rabbit monoclonal anti-HA-Tag (Cell Signaling, 3724S, clone C29F4, 1:500), Rabbit monoclonal anti-Cre Recombinase (Cell Signaling, 12830S, clone D7L7L, 1:500), Mouse monoclonal anti-GAPDH (Sigma-Aldrich, G8795, clone GAPDH-71.7, 1:5000), Mouse monoclonal anti-β-actin (Sigma-Aldrich, A2228, clone AC-74,1:5000), Rat monoclonal anti-DRD1 (Sigma-Aldrich, D2944, clone 1-1-F11 s.E6, 1:500), Mouse monoclonal anti-CB1 (Synaptic systems, 258011, clone 289c1, 1:500), Rabbit polyclonal anti-TH (Pel-Freez Biologicals, P40101, 1:2500), Mouse monoclonal anti-TH (ImmunoStar, 22941, 1:1000), Chicken polyclonal anti-TH (Aves Labs, TYH, 1:500), Chicken polyclonal anti-GFP (Aves Labs, GFP-1020, 1:1000), Rabbit polyclonal anti-RFP (Rockland, 600-401-379, 1:1000), rabbit monoclonal anti-HA-Tag (Cell Signaling, 3724S, clone 29F4, 1:100), Guinea pig polyclonal anti-NeuN (Synaptic systems, 266 004, 1:1000), rabbit polyclonal anti-Iba1 (Wako, 019-19741, 1:1000), and mouse monoclonal anti-TOM20 (Santa Cruz, sc-17764, clone F-10, 1:200).
Validation	The specificity of anti-DAGLA and DAGLB was validated by western blot with N2a cell or primary neuronal cultures infected with Dagla and Daglb-specific gene knock-down vectors. DAGLB and CB1 antibodies were also verified by western blots or immunostaining of tissues from DAGLB and CB1 knockout mice, respectively. The specificity of other antibodies was verified through comparing with previous published studies, based on the molecular weight and staining pattern.

Eukaryotic cell lines

Policy information about cell lines

Cell line source(s)	Neuro-2a (N2a) cells were obtained from ATCC (https://www.atcc.org/products/ccl-131). Human dermal fibroblasts were derived from skin biopsies from affected individuals and age and sex-matched non-neurological controls through standard techniques
Authentication	We purchased the cell line from ATCC and the cell showed the typical size and morphology as expected from N2a cells.
Mycoplasma contamination	All cell lines were tested negative for mycoplasma contamination.
Commonly misidentified lines (See ICLAC register)	No commonly misidentified lines were used in this study.

Animals and other organisms

Policy information about studies involving animals; ARRIVE guidelines recommended for reporting animal research

Laboratory animals	C57BL/6J (#000664), DATIRESCre (#006660), Ai95 (RCL-GCaMP6f) (#028865), and Ai9 (RCL-tdT) (#007909) mice were purchased from the Jackson laboratory. Cnr1loxP/loxP mice were generously provided by Dr. Josephine M. Egan of NIA. Daglb germline KO mice were generously provided by Dr.Ku-Lung Hsu of University of Virginia.The animal age was ranged from embryonic day 16.5 to 20-month-old. Both males and females were used.
Wild animals	No wild animals were used.
Field-collected samples	No field-collected samples.
Ethics oversight	All mouse studies were in accordance with the guidelines approved by Institutional Animal Care and Use Committees (IACUC) of the National Institute on Aging (NIA), NIH.

Note that full information on the approval of the study protocol must also be provided in the manuscript.

Human research participants

Policy information about studies involving human research participants

Population characteristics	Participants were recruited at Xiangya Hospital, Central South University between October 2006 and January 2019 and other hospitals of Parkinson's Disease and Movement Disorders Multicenter Database and Collaborative Network in China (PD-MDCNC, http://pd-mdcnc.com:3111/) established by our group. These participants include 171 probands with ARPD [86 men (50.29%), mean age at onset: 55.55±11.39], 1,571 cases of sporadic EOPD [865 men (55.06%); mean age at onset, 44.00 ±5.57], and 2,152 matched healthy control participants (mean age, 61.78±11.70).
Recruitment	Participants were recruited at Xiangya Hospital, Central South University between October 2006 and December 2021, and other hospitals of Parkinson's Disease and Movement Disorders Multicenter Database and Collaborative Network in China (PD-MDCNC, http://pd-mdcnc.com). We recruited those participants all round the China. We selected the participants with early onset Parkinson disease (EOPD) since EOPD are more likely linked to genetic predisposition.
Ethics oversight	All investigations were conducted according to the Declaration of Helsinki, and the study was approved by the Institutional Review Boards of the Ethics Committee of Xiangya Hospital, Central South University.

Note that full information on the approval of the study protocol must also be provided in the manuscript.
